# Elaboration of an innovative plant biomaterial for its valorization in the treatment of wastewater

**DOI:** 10.1186/s40643-024-00774-4

**Published:** 2024-06-07

**Authors:** El Mokhtar Saoudi Hassani, Imane Mehdaoui, Dounia Azzouni, Rachid Mahmoud, Abdeslam Taleb, Gezahign Fentahun Wondmie, Ahmad Mohammad Salamatullah, Mohammed Bourhia, Samir Ibenmoussa, Mustapha Taleb, Zakia Rais

**Affiliations:** 1 Laboratory of Engineering Electrochemistry, Modeling, and Environment, Department of Chemistry, Faculty of Sciences Dhar Mahraz, Sidi Mohamed Ben Abdellah University, Fez, Morocco; 2 Laboratory of Water and Environmental Engineering, Faculty of Sciences and Techniques of Mohammedia, Hassan II University of Casablanca, 28806 Mohammedia, Morocco; 3 Department of Biology, Bahir Dar University, P.O.Box 79, Bahir Dar, Ethiopia; 4 Department of Food Science & Nutrition, College of Food and Agricultural Sciences, King Saud University, 11, P.O. Box 2460, Riyadh, 11451 Saudi Arabia; 5 Laboratory of Biotechnology and Natural Resources Valorization, Faculty of Sciences, Ibn Zohr University, 80060 Agadir, Morocco; 6 Laboratory of Therapeutic and Organic Chemistry, Faculty of pharmacy, University of Montpellier, 34000 Montpellier, France

**Keywords:** Material, Aleppo pine, Physicochemical characterization, Spectroscopic characterization

## Abstract

The global scientific community is deeply concerned about the deterioration of water quality resulting from the release of industrial effluents. This issue is of utmost importance as it serves to safeguard the environment and combat water pollution. The objective of this work is to elaborate a biomaterial of vegetable origin, based on the twigs of Aleppo pine, and to use it as an abundant and less expensive material for the treatment of wastewater. For this reason, the twigs were treated physically to get the powder called biomaterial FPA (Aleppo pine fiber), which was characterized by physicochemical, and spectroscopic analyses namely scanning electron microscopy (SEM), Fourier transform infrared spectroscopy (FTIR) and X-ray diffraction (XRD). The crystallinity index of FPA was evaluated by the peak height method. The findings indicate that the FPA powder has an acidic nature, exhibiting a porous structure that promotes the adsorption and binding of molecules. Additionally, it has a zero charge point of 5.8 and a specific surface area of 384 m^2^.g^−1^. It is primarily composed of hydroxyl, carboxyl, and amine functional groups, along with mineral compounds and organic compounds, including cellulose and other mineral elements such as Ca, Mg, Fe, Na, P, Al, K, Ni, and Mo. Combining these characteristics, FPA biomaterial has considerable potential for use as an effective adsorbent biomaterial for various wastewater pollutants. Its abundance and relatively low cost make it an attractive solution to the growing challenges of water pollution worldwide.

**Graphical Abstract:**

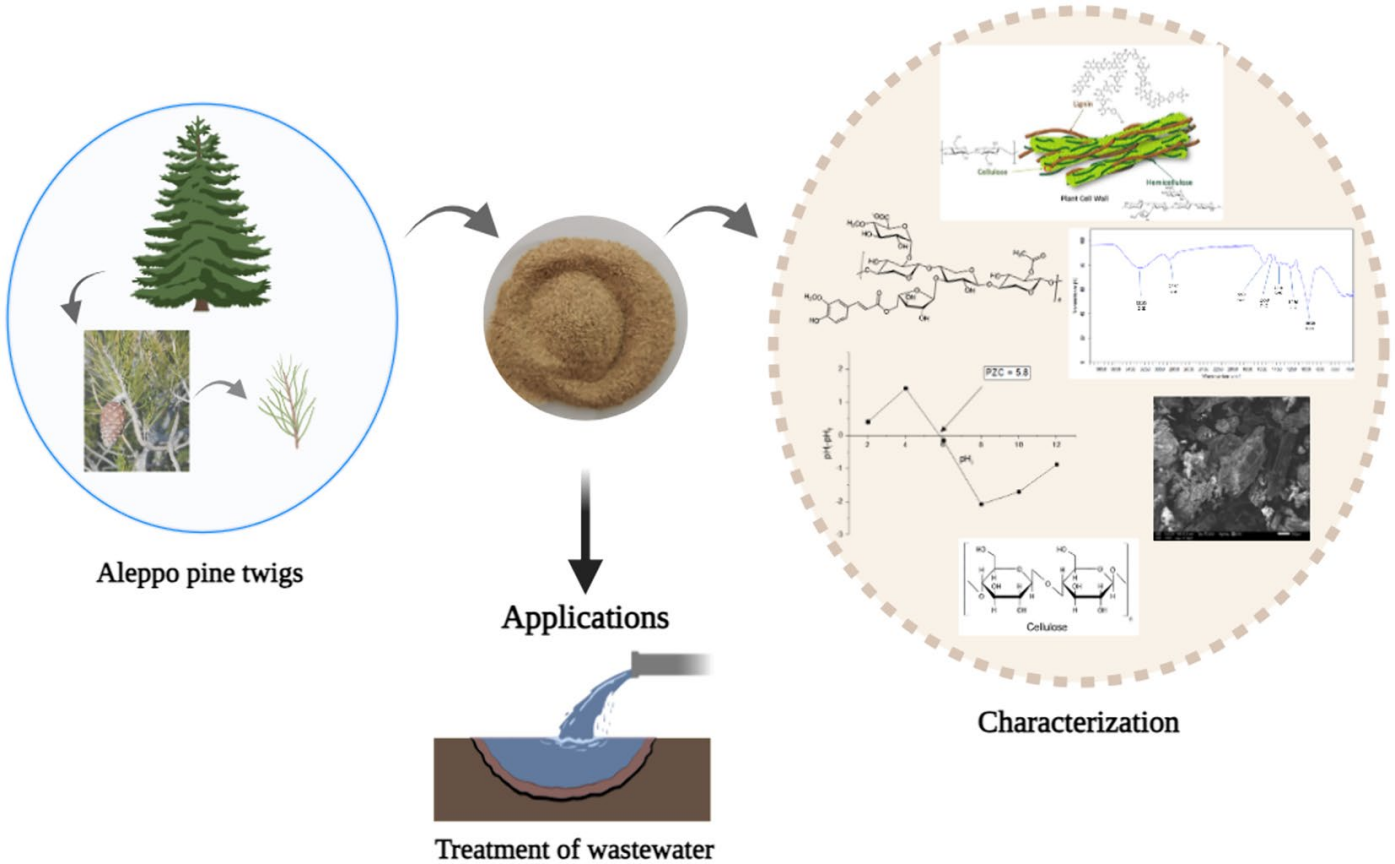

## Introduction

Demographic growth and changing lifestyles are among the biggest contributors to environmental pollution today. **(**Henderson and Loreau 2023**)**. With a current population of 7.4 billion people **(**Leridon 2020**)**, our planet is expected to accommodate nearly 8 billion people by 2030 and forecasters predict between 9 and 10 billion people by 2050 (Randers 2012), which necessarily leads to an increase in water needs.

As to the United Nations, our water requirements are projected to rise by 50% by 2030 (Arora and Mishra 2019). Water contamination is an increasingly significant environmental danger (Rathi et al. 2021), hence highlighting the quality and accessibility of water resources as a prominent problem in the current century (Mishra et al. 2021). Improved management of pollutants, particularly those originating from human activities, is necessary for the preservation of the environment. These pollutants are often defined by the presence of microorganisms, chemical compounds, or industrial waste (Rahman 2020).

Across the globe, almost 80% of wastewater is discharged into the environment without passing through any treatment process. This untreated discharge results in the dispersal of various harmful contaminants into ecosystems, directly impacting the quality of available water resources (Dimé et al. 2020). This reality underlines the urgent need for wastewater treatment systems to prevent pollution and protect the health of aquatic ecosystems and the quality of drinking water.

Treating polluted wastewater is a major challenge for the preservation of the environment and public health. With this in mind, the use of biomaterials offers a promising and sustainable solution. Integrated into various treatment processes, whether of plant or non-plant origin, these biomaterials include oyster shells (Kim et al. 2020), activated carbon (Jjagwe et al. 2021), biological membranes (Alfonso-Muniozguren et al. 2021), biochars (Kamali et al. 2021), as well as biomaterials derived from natural wastes such as wood residues (Jiao et al. 2020), vegetable waste (Matei et al. 2021) and synthetic organic materials (Saoudi Hassani and al. 2024) (Azzouni et al. 2022). Acting as decontamination agents, they capture contaminants and facilitate their removal from wastewater. In addition to contributing to water decontamination, their use enables sustainable waste recovery, reducing the environmental footprint of wastewater treatment facilities and offering economically viable solutions. This integrated approach, combining purification efficiency and resource recovery, is an important step towards more sustainable management of our water resources and the preservation of aquatic ecosystems.

In light of the ongoing quest for cost-effective and sustainable materials for wastewater treatment, this study focuses on the development of an innovative plant-based biomaterial from Aleppo pine fibers, an abundant and low-cost natural resource. The primary objective of this research is to determine the physicochemical and spectroscopic properties of this newly developed biomaterial in order to gain a comprehensive understanding of its structure and adsorption capacities. This in-depth understanding is crucial for assessing its potential effectiveness in wastewater treatment, particularly its ability to remove contaminants and pollutants from effluents. By recommending the use of this biomaterial as a wastewater treatment material, this study paves the way for practical, eco-friendly, and economically viable solutions to address the global challenge of water pollution. By harnessing renewable natural resources and offering sustainable alternatives to traditional methods of wastewater treatment, this research contributes to building a cleaner and more environmentally friendly future while providing economic opportunities for local communities.

The pine is a conifer of the Pinus genus, belonging to the Pinaceae family, which is distributed throughout the world (Lazreg et al. 2018). It is found mainly in North Africa and Spain. It likes the cold-temperate climate of the northern hemisphere, where it occupies all stages of vegetation. In Morocco, among the species of the genus Pinus, we find the Aleppo pine, which occupies an area of 65,000 hectares and is present in the Rif, especially on the Mediterranean slope, the central Middle Atlas, and some valleys of the High Atlas, and represents a natural resource to be developed.

## Materials and methods

### Preparation of the biomaterial

**Fig. 1:**
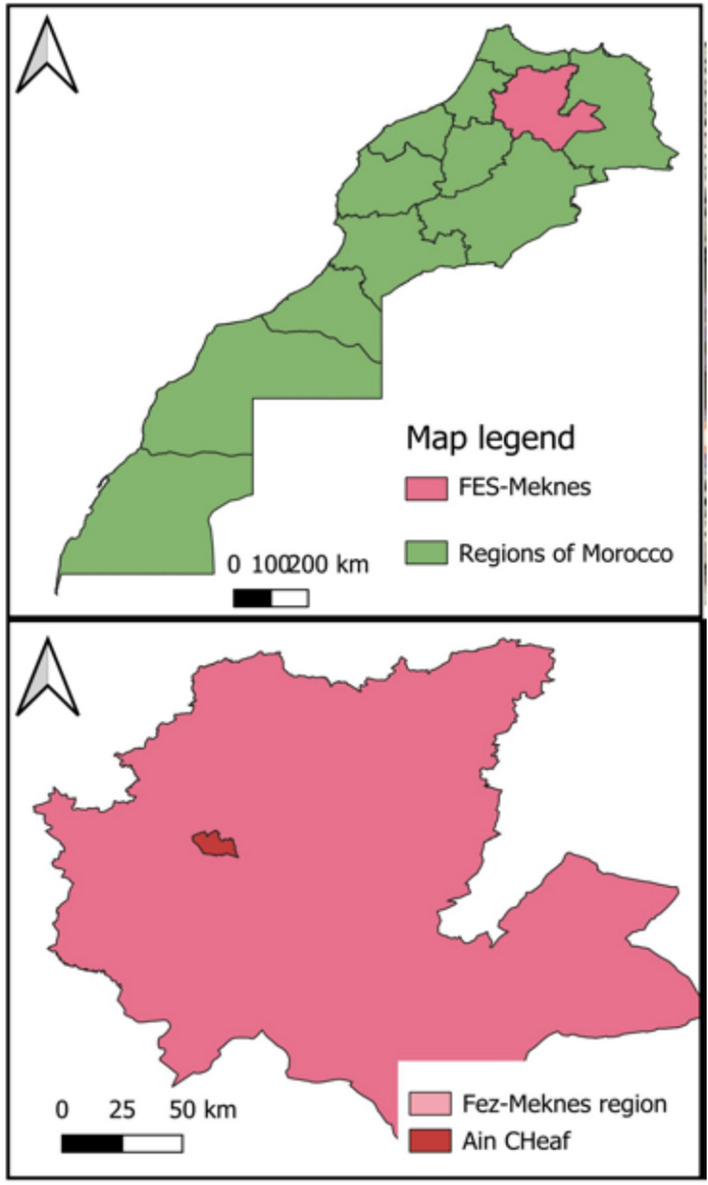
Geographical location of the Ain Chkef forest - Fez - Morocco

The Aleppo pine fiber powder studied in this work was obtained from pine *(Pinus halepensis)* twigs that were collected from the Ain Chkef forest in the city of Fez, Morocco (Fig. 1). This forest has an area of 46 hectares and represents a mosaic of carob trees, acacia cyanophylla, eucalyptus, and mainly Aleppo pine.

**Fig. 2:**
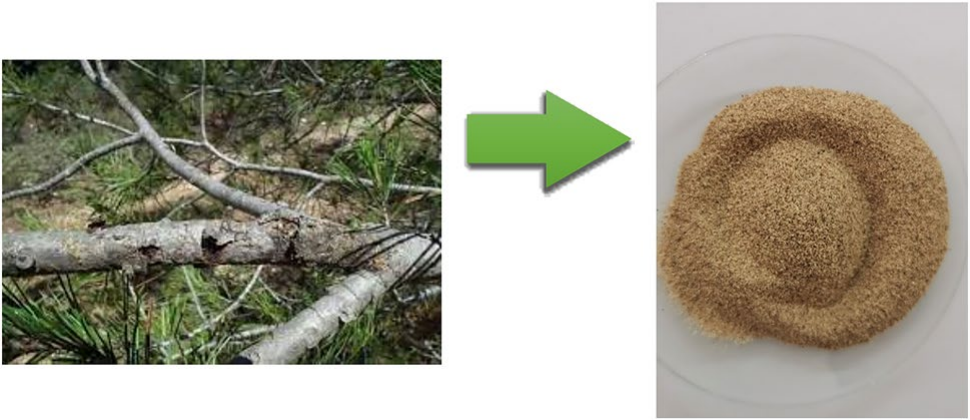
Preparation of the FPA biomaterial

The Aleppo pine twigs were harvested before being subjected to a rigorous treatment process as follows:

First, they were carefully washed with distilled water to remove residue. Next, they were placed in an oven and dried at a constant temperature of 60 °C for 48 h. Once dried, the twigs were crushed and sieved using a 1 mm diameter sieve. This meticulous process resulted in the formation of a fine powder called FPA biomaterial, characterized by particle sizes of less than 1 mm (Fig. 2).

To confirm its properties, the FPA biomaterial was subjected to extensive analysis, including detailed physicochemical and spectroscopic analyses. These analytical methods enabled precise identification of its composition and characteristics, providing a solid basis for its future use in the treatment of polluted water.

### Characterization of the FPA biomaterial

#### Physicochemical analysis

They consist of measuring pH, electrical conductivity (EC), organic matter (OM), total organic carbon (TOC), and chemical composition of elements and mineral oxides that have been established according to standardized norms (Rais et al. 2017).

The Hanna 209 pH meter (ISO 10,390 − 2005) was used to measure the pH, whereas the HANNA EC214 conductivity meter (ISO 11,256 − 1994) was used to assess the electrical conductivity (EC). The determination of moisture and dry matter was conducted by measuring the mass loss due to water evaporation using a BOXUN-type oven (ISO 11,465 − 1993). The organic matter (OM) was processed by calcination at a temperature of 550 °C (NF EN 13,039 − 2011) using a Barnstead Thermolyne 1400 °C muffle furnace. The OM value was used to determine the mineral matter (NF EN 13,039 − 2011) and total organic carbon (ISO 14,235 − 1998).

The concentration of mineral elements in our FPA biomaterial has been assessed using the ICP (Inductively Coupled Plasma) technique, which enables precise analysis of mineral components. This method uses high-temperature plasma to ionize samples, enabling elemental concentrations to be measured with high sensitivity and a wide detection range.

The point of zero charge (PZC) was determined by the solid addition method, which consists of stirring solutions composed of 5 g of biomaterial in 150 mL of NaCl (0.05 M) solution for 48 h, the initial pH (pH_i_) of which was adjusted by adding HCl or KOH (0.1 M). After decantation, the final pH values of the supernatants (pH_f_) were measured. The difference between the initial and final pH values (ΔpH = pH_i_ - pH_f_) was plotted against pHi. The point of intersection of the resulting curve at ΔpH = 0 corresponds to the pH of the zero-charge point PZC.

The specific surface area of FPA biomaterial was determined by the methylene blue adsorption method (Dhorabe et al. 2015). From the maximum adsorption capacity Q_mL_ (mg/g), the estimation of the specific surface area SL is given by equation (Eq. 1):

1 $${\text{S}}_{{\text{L}}} {\text{ = Q}}_{{{\text{mL}}}} {\text{ x S}}_{{{\text{BM}}}} {\text{ x N}}_{{\text{A}}}$$

With:

S_BM_ = 175.10^−20^ m^2^: the surface occupied by a methylene blue molecule.

N_A_ = 6.022.10^23^ mol^−1^: the Avogadro´s number.

The functional groups present on the surface of the FPA biomaterial were analyzed using the Boehm method, which characterizes the acidic functional groups present on the surface of materials by reacting them with Lewis bases such as sodium or potassium hydroxides, thus forming salts. By titrating the resulting solution with a known acid solution, such as hydrochloric acid, the concentration of these acid groups can be determined (Boehm 2002). This process provides valuable information on the nature and quantity of acidic functional groups in the material, essential for understanding its chemical properties and behavior in various applications.

#### Spectroscopic analysis

These analyses include the Fourier Transform Infrared Spectroscopy (FTIR) which was performed by FTIR spectrophotometer Bruker (Germany), model Vertex 70, in order to determine the functional groups that compose it (Servant et al. 2011), X-ray diffraction (XRD) performed, using a PAN-CriticalX’Pert Pro X-ray diffractometer equipped with a monochromatic Cu-Kα source (1.54 Å), in order to identify the crystalline phases present in the FPA (« Serna et al. 2014 », s. d.), thermogravimetric analysis (TGA) by a LINSEIS STA PT1600 Thermogravimetric Analyzer, under nitrogen atmosphere and a temperature range that extends from 0 to 1000 °C, in order to observe the thermal decomposition effects of the biomaterial and qualify its stability, and scanning electron microscopy coupled with an EDX probe (SEM-EDX), model QUANTA 200 to study the surface morphology of the FPA biomaterial (Soares et al. 2017).

## Results and discussion

### Physicochemical characterization of the FPA biomaterial

The obtained results showed that FPA biomaterial elaborated in the present study possesses promising physicochemical characteristics and can be used as ecofriendly agents for the treatment of polluted water. The helicoidal, fibrous structure of FPA, described in Tables 1 and 2, presents favorable characteristics for pollutant adsorption. Its slightly acidic pH, tending towards neutrality, indicates compatibility with a wide range of treatment conditions. In addition, its low conductivity and high specific surface area of 384 m^2^/g, compared with other biomaterials in the literature (Table 3), make it an attractive candidate for contaminant adsorption. The biomaterial’s predominantly organic composition, with a minor presence of mineral elements including Ca, Mg, Fe, Na, P, Al, K, Ni, M…, offers varied possibilities for interaction with pollutants present in water.

**Table 1:** Physico-chemical characteristics of FPA biomaterial

Parameters	pH	CE (µs. cm^−1^)	H (%)	MS (%)	MO (%)	*P* (mg/g)	COT (%)	NTK (%)	C/*N*	Ssp m^2^.g^−1^
Values	6.26	500	9.81	90.19	0.73	0.5454	57.58	1.435	40.12	384

**Table 2:** Composition of the FPA in chemical elements

Elements	Al	As	Ba	Ca	Mo	Cr	Cu	Fe	K	Se	Mg	Mn	Na	Ni	Si	Zn
Concentration (mg/g)	0.252	< 0.01	< 0.01	2.221	0.013	< 0.01	< 0.01	0.699	0.192	< 0.01	1.043	< 0.01	0.695	0.034	< 0.01	< 0.01

**Table 3:** Comparison of specific surfaces for different biomaterials

Biomaterial	Swine manure biochar	Bottom ash	Date pits	Crab shell	Orange peels	Banana peels	FPA
SSP (m^2^/g)	198.8	2.1	4.039	191	2.341	21.46	384
References	(Wang et al. 2020)	(El mouhri et al. 2021)	(Hassan et al. 2020)	(Francis et al. 2021)	(Kamsonlian et al. 2013)	(Temesgen et al. 2018)	–

**Fig. 3:**
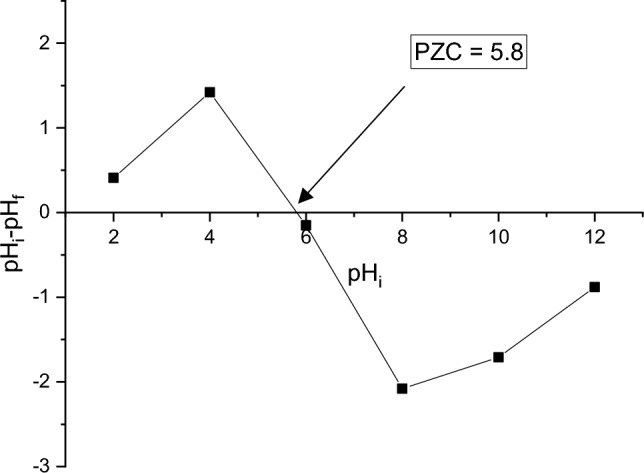
Zero charge point of the powder (FPA)

Zero charge point analysis, as depicted in Fig. 3 for FPA biomaterial, is of key importance in suggesting this material for the treatment of polluted water. By identifying the pH at which the FPA surface presents a net zero electrical charge, this analysis provides crucial clues as to its adsorption behavior towards pollutants. When the pH is below the isoelectric point of 5.8, the FPA displays a positive charge, making it suitable for adsorption of negatively charged ions present in polluted water. This ability offers a valuable opportunity to remove certain types of contaminants such as heavy metals and pesticides. Similarly, beyond this threshold, as the surface charge becomes negative, the material becomes more capable of adsorbing positively charged dyes and compounds, broadening its spectrum of action in the treatment of polluted water. By understanding these variations in charge as a function of pH, we can propose more effective and specific treatment conditions, enabling more selective elimination of pollutants and an overall improvement in the efficiency of the depollution process.

**Table 4:** FPA biomaterial surface functional groups

Functional groups	Carboxyls (COOH)	Hydroxyls (OH)	Lactones (C = O)
Quantity (mmol/g)	0.233	0.343	0.198

The results of the functional groups (Table 4) show that the FPA biomaterial consists mostly of hydroxyl functional groups with a value of 0.343 mmol.g^−1^, carboxyl with a value of 0.233 mmol.g^−1^, and lactone with a value of 0.198 mmol.g^−1^, this explains the slightly acidic surface of the FPA, previously predicted from the results of the zero charge point.

### Spectroscopic characterization of the FPA biomaterial

#### Infrared spectroscopy (FTIR)

**Fig. 4:**
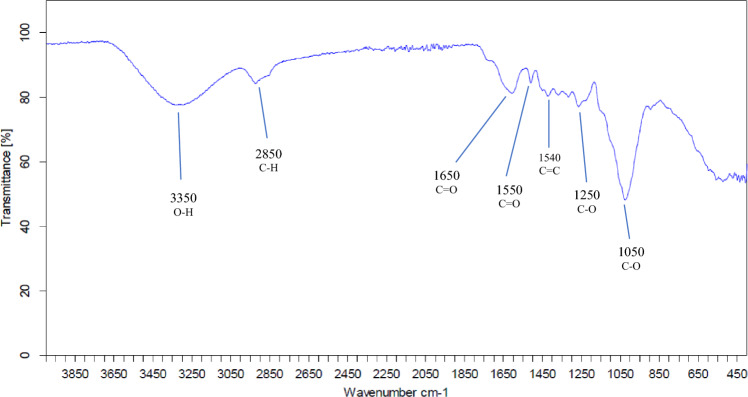
Infrared spectrum of FPA biomaterial

The presence of several chemical functionalities, namely O–H, C–H, C = O, C = C, and C–O, is confirmed by the infrared spectrum of the FPA biomaterial (see Fig. 4). The O-H stretching vibration is associated with a bond at 3301.17 cm^−1^. The formation of this bond is likely ascribed to the presence of alcohol and phenol groups, which are inherent to cellulose and lignin, which are recognized as significant components of plants (Barhoum et al. 2020).

The band observed around 2850 cm^−1^ is probably related to the symmetric and asymmetric stretching of the aliphatic C–H chains, especially due to the presence of the methyl (CH_3_-) and methylene (-CH_2_-) groups of the lateral chains. The peaks around 1650 and 1550 cm^−1^ could be attributed to the C = O vibration due to the carboxylic and carboxylate form of cellulose (Hokkanen et al. 2016) which may act as an active group for binding toxic ions present in water (Lim and Aris 2014). While the peak at 1540 cm^−1^ can be attributed to the presence of aromatic rings with C = C bonds and to lignin. The peaks at 1250 and 1050 cm^−1^ can correspond to ether and C–O alcohol, especially in cellulosic compounds.

**Fig. 5:**
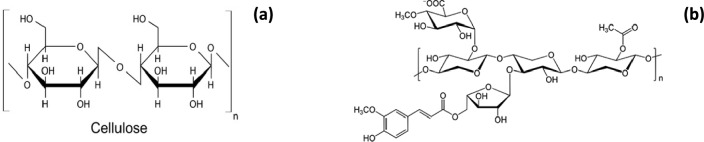
Chemical structure of cellulose (**a**) and hemicellulose (**b**)

The results of the infrared spectroscopy align with those obtained previously in determining the functional groups, thus confirming the presence of Carboxyl (COOH), Hydroxyl (OH), and Lactone (C = O) groups on the surface of the FPA biomaterial. These functional groups are the main characteristic motifs of cellulose and hemicellulose (Fig. 5).

The Chemical functions such as carboxyl (COOH), hydroxyl (OH) and lactone (C = O) present on the surface of FPA biomaterial act as complex adsorption sites, facilitating the selective capture of pollutants from wastewater. They establish chemical bonds with pollutant particles through mechanisms such as electrostatic interactions and hydrogen bonding, thus contributing to water purification. The carboxyl group is capable of forming chemical bonds with metal ions, thus promoting their capture. Hydroxyl groups interact with pollutants containing electrophilic functional groups, while lactone can capture pollutants containing nucleophilic functional groups. These interactions enable selective adsorption of pollutants, improving the effectiveness of FPA biomaterial treatment of polluted water.

**Fig. 6:**
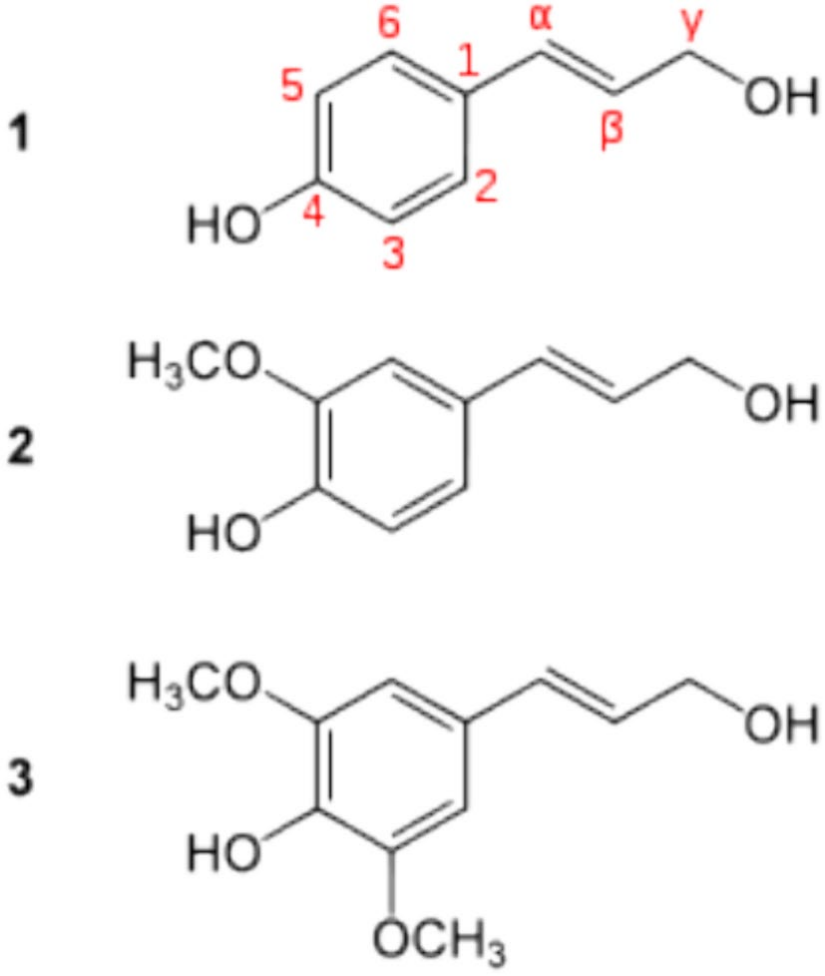
The three main monolignols that give rise to lignin: 1 p-coumaryl alcohol; 2 coniferyl alcohols; 3 sinapyl alcohols

The results of research carried out on FPA have shown that it is generally composed of cellulose, hemicellulose, and lignin and includes mainly functional groups such as ketones, hydroxyl, carboxyl, and phenol (Figs. 5 and 6) that are strongly involved in the bonds between polluting particles, which advocates their use as an adsorbent for the treatment of polluted wastewater.

#### Scanning electron microscope

**Fig. 7:**
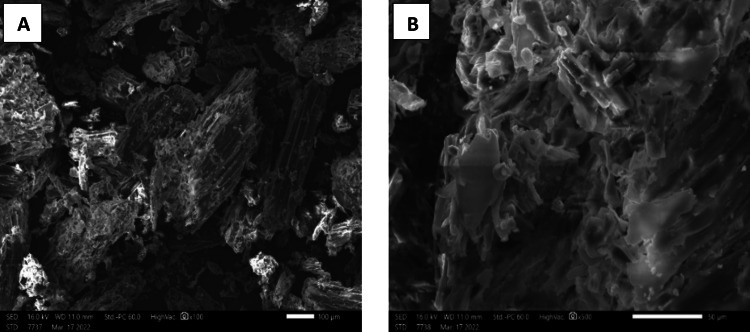
Scanning electron micrograph showing the porosity of the FPA powder surface at different magnifications (**A** x100, **B** x500)

The FPA particles have a distinctive morphology characterized by condensed agglomerates, giving their structure significant porosity down to a depth of around one millimeter. In addition, their rough texture and porous surface further enhance their ability to adsorb pollutants. These characteristics are favorable factors for the adsorption of contaminants by an ion exchange process involving the attachment of cationic or anionic particles. Consequently, the distinctive morphological and structural properties of FPA play a crucial role in its effectiveness as a depollution agent in the treatment of contaminated water. The findings presented here as shown in Fig. 7 are consistent with the results reported in prior research conducted by (Bozbaş and Boz 2016) and (Aditya and Hossain 2018).

**Fig. 8:**
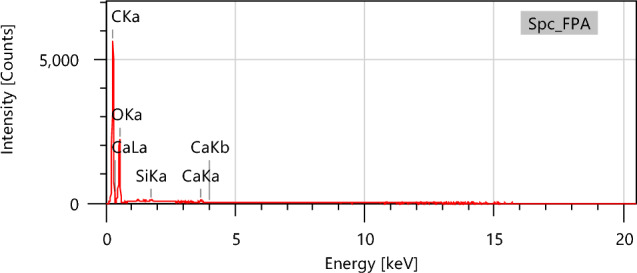
EDX spectrum of the FPA biomaterial

By using energy-dispersive EDX analysis, we were able to provide a quantitative assessment of the chemical constituents present in the FPA, as seen in Fig. 8. The EDX results shown in Table 5 reveal that carbon and oxygen make up around 99.45% of the weight of FPA, whereas calcium and silicon are present in smaller amounts.

**Table 5:** Percentages of weight and atom content of the different elements contained in the FPA biomaterial

Element	Mass %	Atom %
C	57.80 ± 0.13	64.83 ± 0.14
O	41.45 ± 0.30	34.90 ± 0.25
Si	0.14 ± 0.02	0.07 ± 0.01
Ca	0.60 ± 0.03	0.20 ± 0.01
Total	100.00	100.00

#### Thermogravimetric ATG-ATD analysis of FPA

**Fig. 9:**
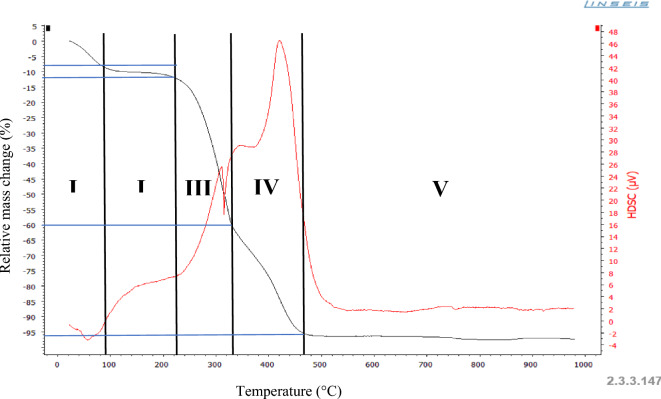
Thermograph of the FPA biomaterial

Thermogravimetric analysis (ATG) was used to assess the thermal stability of FPA. The results, shown in Fig. 9, indicate that FPA biomaterial undergoes four distinct stages of thermal decomposition when processed between 30 and 1000 °C. These stages are characterized by four inflection points at specific temperatures: T1 = 90 °C, T2 = 220 °C, T3 = 330 °C and T4 = 465 °C. At 1000 °C, only 4% of the initial biomaterial remains, confirming its organic nature.

Indeed, the first loss of (-8%) is located around 90 °C accompanied by an endothermic peak, which corresponds to the loss of water physically adsorbed by FPA (Bouchair and Bouremmad 2019). A second slight mass loss (− 4%) appears in the 90 to 220 °C range attributed to the decomposition of hemicellulose characteristic of wood compounds (Haddad 2018). The third mass loss is of the order of − 60% in the range 220–330 °C and is accompanied by an exothermic peak. This loss may correspond to the decomposition of cellulose (Haddad 2018), while the last loss (-96%) located between 330 °C and 465 °C may be related to the degradation of lignin (Haddad 2018).

**Fig. 10:**
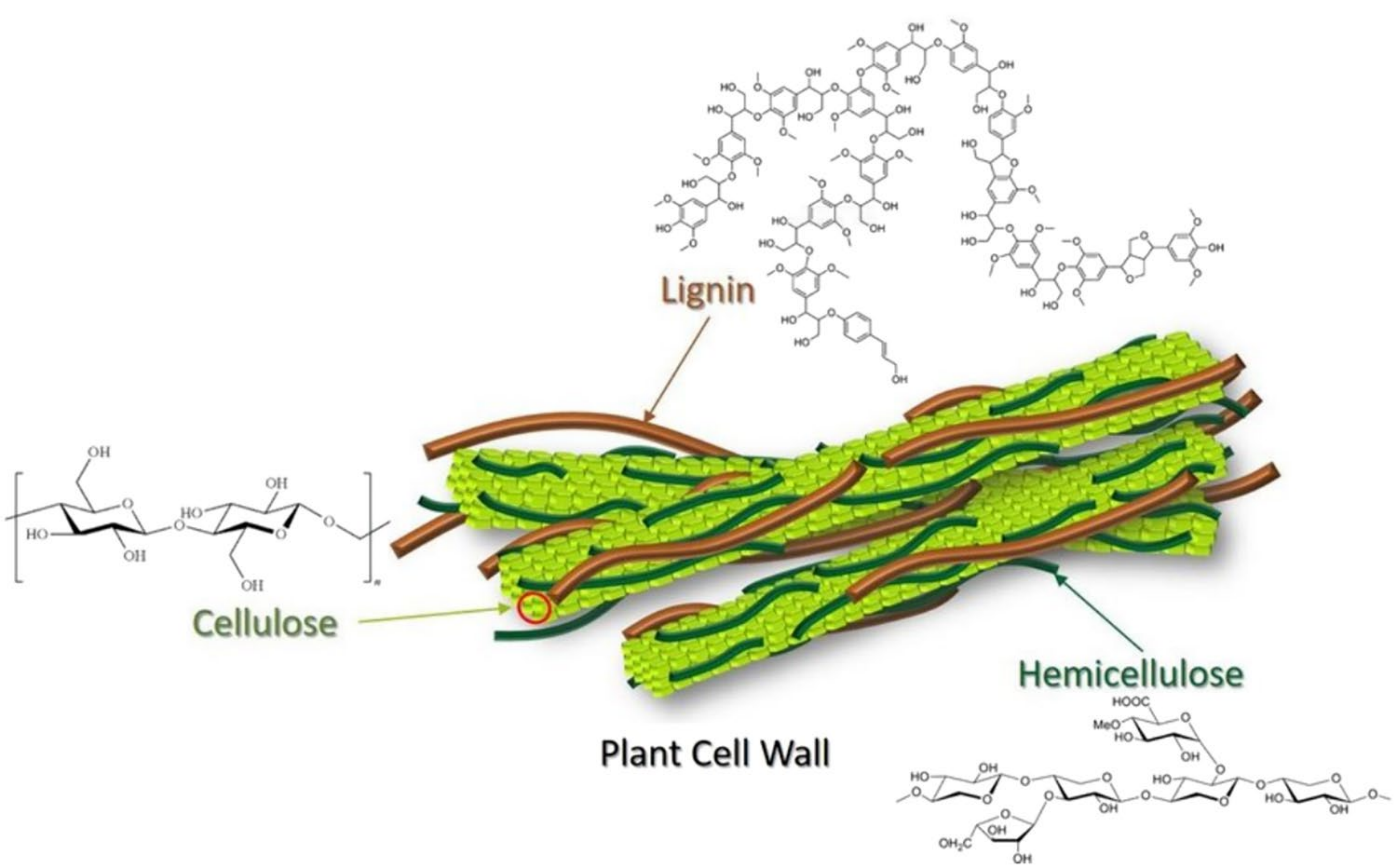
Main constituents structure of lignocellulosic compounds (Jensen et al. 2017)

This important weight loss reflects the nature and composition of the FPA biomaterial in organic components: lignin, cellulose, and hemicellulose characteristic of the lignocellulosic biomass which are major polymeric components of biomass materials and vary considerably depending on the type of biomass. Figure 10 shows the mechanical and chemical structure of lignocellulosic biomass.

The value of this analysis lies in its ability to predict the suitability of this biomaterial as an adsorbent, particularly for high-temperature industrial effluents such as those from the phosphate industry, textiles, steelworks, foundries and rolling mills. These effluents may experience a reduction in the mass of our FPA biomaterial (as shown in Fig. 9) during the treatment process, due to the high temperature of the effluent. This mass reduction can be the result of various processes, such as thermal degradation or volatilization of adsorbed organic compounds. Understanding how FPA reacts at these elevated temperatures is crucial to ensuring its continued effectiveness as an adsorbent in demanding industrial environments. Using the data from this analysis, treatment parameters can be adjusted to optimize the use of FPA in these specific applications, thus ensuring effective and sustainable depollution of industrial effluents (Saoudi Hassani et al. 2024).

#### X-ray diffraction (XRD) of the FPA biomaterial

The X-ray diffractogram of FPA is presented in Fig. 12. The major peaks displayed in Table 6, located at 2θ angles of 22.0224°, 31.7726°, 43.4867°, 50.6576°, and 72.5276°, are indicative of cellulose I characteristics. (Manju et al. 2022). Indeed, natural cellulose is known to be a mixture of two different crystalline forms, namely Iα (triclinic) and Iβ (monoclinic) celluloses, whose fractions vary depending on the origin of the cellulose sample (Sang et al. 2022; Jang et al. 2023).

The (200) plane of the crystalline phase of cellulose is responsible for the biggest peak found at 2θ = 22.21°. Adjacent to this stage, there is an amorphous phase situated at 2θ = 18.28°, mostly composed of lignin, which is linked to the cell wall. This contributes to the wood’s mechanical strength. According to (Sebayang and Hasan 2012), a reduction in its concentration results in an augmentation of the crystalline percentage.

**Fig. 11:**
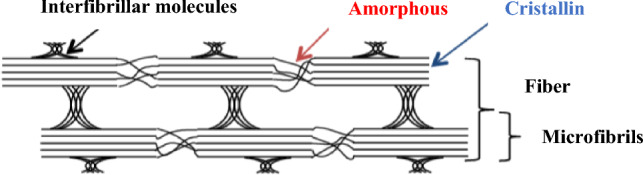
Composition of a cellulosic fiber

Figure 11 illustrates the spatial distribution of the crystalline phase and the amorphous phase within a cellulose fiber. The crystalline phase is characterized by an orderly and regular arrangement of cellulose molecules, while the amorphous phase exhibits a less structured and more random organization.

**Fig. 12:**
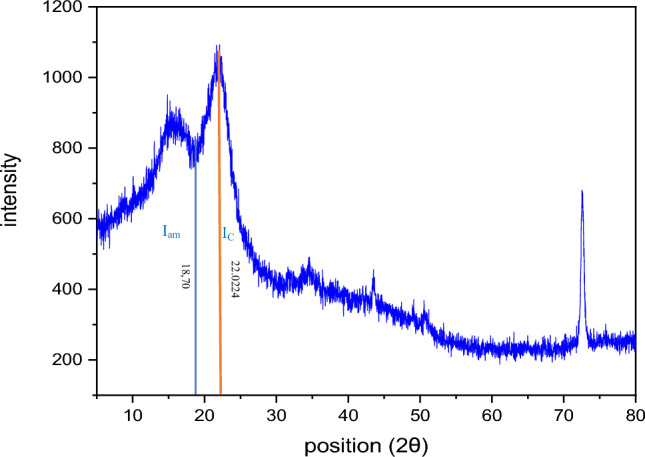
X-ray spectrum of the biomaterial (FPA)

The peak height method, also known as the Segal method, is the most widely used analytical approach to characterize the crystallinity of cellulosic samples (Segal et al. 1959). It is the ratio of the intensity of the crystalline cellulose (I_c_-I_am_) to the total intensity (I_c_) at 2θ = 22.0224°, where I_am_ is the intensity of the amorphous cellulosic phase at 2θ = 18.28° that corresponds to the minimum position of the diffraction pattern. Figure 12 shows an example of the crystalline and amorphous peaks used in Eq. 2:

2 $$CrI\left(\%\right)=\frac{{I}_{C}-{I}_{am}}{{I}_{C}}\times 100$$

The average size of the crystal units was calculated from the Scherrer equation (Eq. 3) (Ven and Godbout 2013). The method is based on the half-value width of the diffraction patterns obtained in the X-ray reflected crystal region. The crystallite size (Lhkl) was determined using the diffraction pattern (Fig. 12).

3 $${L}_{hkl}=\frac{k\lambda }{{\upbeta }\text{c}\text{o}\text{s}{\uptheta }}$$

where k is the Scherrer constant whose value is 0.94, λ is the wavelength of the X-ray (0.154 nm), β in radians is the total width of the diffraction peak and θ is the corresponding Bragg angle.

The results reveal that the most distinct crystalline peak is that corresponding to the (200) reflection, suggesting strong crystalline organization along this direction. This crystalline peak (200) is particularly significant as it is directly associated with crystallite size, which was determined at a wavelength of 1.71 nm. This observation highlights the importance of the (200) crystalline peak in determining the lateral dimension of the crystallinity of the material studied.

**Table 6:** List of FPA crystal peaks

	Pos. (°2Th)	Height	β (rada)	Crl (%)	L_hkl_ (nm)
1	22.0224	245.29	0.081	27%	1.71
2	31.7726	27.25	–		–
3	43.4867	55.68	–		–
4	50.6576	26.28	–		–
5	72.5276	414.95	9.198 × 10^−3^		15.05

## Conclusion

The characterization of the FPA has significant importance in order to anticipate its possible use in wastewater treatment. This study demonstrates that the powder derived from Aleppo pines has a mild acidity that leans towards neutrality. Additionally, it possesses low conductivity, as seen by its specific surface area of 384 m^2^/g and a PZC value of 5.8. The powder is mostly made of organic matter and contains a minimal proportion of mineral matter.

It is non-toxic, with carbonyl, hydroxyl, carboxyl, amine, carbonate, and aromatic functional groups. Its texture is porous and has a low crystallinity related to the presence of cellulose and lignin. These results obtained in this study can be considered encouraging and in line with the circular economy vision for the full valorization of the FPA biomaterial and its use in the removal of pollutants from wastewater, given its abundance, low cost, and easy preparation.

## Statement of Novelty

The novelty and scientific significance of this work is to elaborate on a new biomaterial of vegetable origin, based on the twigs of Aleppo pine, and to study its physicochemical and spectroscopic characteristics in order to use it as an abundant and less expensive material for wastewater treatment.

## Data Availability

Data will be available upon request from the corresponding author.
